# Successful desensitization protocol to alglucosidase and avalglucosidase alfa in a patient with infantile-onset Pompe disease

**DOI:** 10.1016/j.ymgmr.2025.101207

**Published:** 2025-03-20

**Authors:** Miriam Gendive, Teresa C. Delgado, María Unceta, Arantza Arza, Beatriz Sordo, Aritza Segurola, Javier De las Heras

**Affiliations:** a Department of Pediatrics, Araba University Hospital, 01009 Vitoria, Spain; b Biobizkaia Health Research Institute, 48903 Barakaldo, Spain; c IKERBASQUE, Basque Foundation for Science, Bilbao, Spain; d Metabolism Section, Biochemistry Laboratory, Cruces University Hospital, 48903 Barakaldo, Spain; e Pharmacy Service, Cruces University Hospital, 48903 Barakaldo, Spain; f Allergy Service, Cruces University Hospital, 48903 Barakaldo, Spain; g Division of Paediatric Metabolism, CIBERER, MetabERN, Cruces University Hospital, 48903 Barakaldo, Spain; h Department of Paediatrics, University of the Basque Country (UPV/EHU), 48940 Leioa, Spain

**Keywords:** Pompe disease, Infantile-onset Pompe disease, Alglucosidase alfa, Avalglucosidase alfa, Desensitization, Anaphylaxia, Infusion associated reaction

## Abstract

Infantile-onset Pompe disease is a lysosomal disease characterized by cardiomyopathy and muscle weakness that, without specific treatment, is fatal within the first two years of life. We present the case of an infant who developed anaphylaxia to enzyme replacement therapy with alglucosidase-alfa. We provide a desensitization protocol to alglucosidase-alfa and, for the first time, a desensitization protocol to avalglucosidase-alfa, both delivered in a reasonable time of <6 h, and without any further reactions in the patient.

## 1 Introduction

Classic infantile-onset Pompe disease (IOPD) is a rare lysosomal storage disorder characterized by severe hypertrophic cardiomyopathy and profound muscle weakness [1]. It is a severe disease with a rapidly progressive course and, without specific treatment, death occurs within the first 2 years of life [2]. Enzyme replacement therapy (ERT) with alglucosidase alfa and the recently approved molecule avalglucosidase alfa has led to improvements in survival rates and clinical outcomes [3,4]. However, as is the case with other forms of protein-based therapies, infusions of both molecules have been associated with infusion associated reactions (IARs) of variable severity [5].

## 2 Case report

We report a case of a IOPD patient diagnosed in the first days of life. He was the first child of consanguineous parents. He was asymptomatic when an echocardiogram was performed after a heart murmur was heard. The cardiac assessment revealed a severe biventricular hypertrophic cardiomyopathy with normal systolic function. Laboratory analysis showed increased creatine kinase (CK) levels of 1281 U/L. Given the suspicion of IOPD, a dried blood spot sample was immediately sent to a reference laboratory to assess acid alfa-glucosidase (GAA) enzyme activity, and results at 7 days of life were in the diagnostic range of IOPD (0,1 μmol/L/h, reference range 7–37 μmol/L/h). Molecular analysis confirmed a homozygous pathogenic variant (c.1551 + 1G > A) in the *GAA* gene. When the patient was sixteen days of life, specific treatment was started with ERT (alglucosidase alfa, 40 mg/kg every 2 weeks), with an immunomodulation regimen in the ERT-naïve setting irrespective to CRIM status with methotrexate, rituximab and intravenous immunoglobulin [6]. There was a good response to ERT, with the patient scoring within the normal range in Alberta Infant Motor Scale longitudinal assessments, in addition to not requiring any respiratory or nutritional assistance. As expected, hypertrophic cardiomyopathy had resolved by five months of age.

At 18 months of age, the patient presented an urticarial reaction related to the alglucosidase alfa infusion. The infusion was temporarily paused and he was treated with intravenous antihistamine with good clinical response, and the full dose could be delivered. In the next infusion, although the patient was premedicated with antihistamine and antipyretics, and the infusion rate was slowed, the patient presented an urticarial reaction with bronchospasm and upper lip edema. Total IgE level was high (123 KU/L) and the result of an intradermal skin test to alglucosidase alfa (0.5 mg/mL) was positive. Therefore, a 12-step desensitization protocol was prepared to deliver alglucosidase alfa 20 mg/kg/weekly (Table 1A). Dexchlorpheniramine (0.2 mg/kg), methylprednisolone (1 mg/kg) and montelukast (4 mg) were administered as premedication one hour before the start of the drug infusion. Premedication with methylprednisolone was gradually stopped in the patient. The desensitization protocol was successful with no more reactions in the patient.

**Table 1:** Intravenous desensitization protocol to A) alglucosidase alfa; and B) avalglucosidase alfa. NaCL: sodium chloride.

Table 1 A. Age: 18 months. Weight: 10 kg.						
ALGLUCOSIDASE alfa (50 mg = 10 mL) Dose: 20 mg/kg.						
Solution type	Solution content		Concentration (mg/mL)		Volume administered (mL)	Dose administered (mg)
1	4 mL solution 2 + 36 mL 0.9 % NaCl		0.02		9.25	0.185
2	4 mL solution 3 + 36 mL 0.9 % NaCl		0.2		18.75	3.75
3	50 mL drug (250 mg) + 75 mL 0.9 % NaCl		2		98	196
Step	Solution type	Rate (mL/h)	Infusion duration (min)	Volume infused /step (mL)	Dose administered/step (mg)	Cumulative dose (mg)
1	1	2	15	0.5	0.01	0.01
2	1	5	15	1.25	0.025	0.035
3	1	10	15	2.5	0.05	0.085
4	1	20	15	5	0.1	0.185
5	2	5	15	1.25	0.25	0.435
6	2	10	15	2.5	0.5	0.935
7	2	20	15	5	1	1.935
8	2	40	15	10	2	3.935
9	3	5	15	1.25	2.5	6.435
10	3	10	15	2.5	5	11.435
11	3	20	15	5	10	21.435
12	3	30	178.6	89.25	178.5	200
		Total time (min):	343.6		Total Dose: 200 mg	


Table 1 B. Age: 26 months. Weight: 12 kg.						
AVALGUCOSIDASE alfa (100 mg = 10 mL) Dose: 40 mg/kg.						
Solution type	Solution content		Concentration (mg/mL)		Volume administered (mL)	Dose administered (mg)
1	4 mL solution 2 + 36 mL glucose 5 %		0.03		9.25	0.2775
2	4 mL solution 3 + 36 mL glucose 5 %		0.3		18.75	5.625
3	48.6 mL drug (486 mg) + 113.4 mL glucose 5 %		3		158	474
Step	Solution type	Rate (mL/h)	Infusion duration (min)	Volume infused /step (mL)	Dose administered/step (mg)	Cumulative dose (mg)
1	1	2	15	0.5	0.015	0.015
2	1	5	15	1.25	0.0375	0.0525
3	1	10	15	2.5	0.075	0.1275
4	1	20	15	5	0.15	0.2775
5	2	5	15	1.25	0.375	0.6525
6	2	10	15	2.5	0.75	1.4025
7	2	20	15	5	1.5	2.9025
8	2	40	15	10	3	5.9025
9	3	10	15	2.5	7.5	13.3025
10	3	20	15	5	15	28.4025
11	3	40	15	10	30	58.4025
12	3	80	105.4	140.5	421.5	480
		Total time (min):	270.4		Total Dose: 480 mg	

Last biochemical data before the anaphylaxis episode showed a CK level of 860 U/L and a glucose tetrasaccharide (Glc4) level of 57 mmol/mol Cr. As the patient suffered a clinical decline with an increase in CK and Glc4 levels (CK up to 1799 U/L and Glc4 up to 72 mmol/mol Cr), a decision was made to switch treatment to avalglucosidase alfa.

Given the similarity of the two molecules and the positive intradermal skin test to avalglucosidase alfa (0.5 mg/mL) in the patient, a desensitization protocol was prepared for the new treatment administration at a dose of 40 mg/kg/every other week (Table 1B), with clinical and biochemical improvement (last CK level: 413 U/L and last Glc4 level: 37 mmol/mol Cr), and no more IARs so far.

## 3 Discussion

ERT with alglucosidase alfa has greatly improved prognosis in IOPD [3,4]. However, treatment outcome is largely dependent on age at initiation, and clinical response can be influenced by factors like the extent of muscle damage at the start of ERT, CRIM status, immune response and dosage, among others [7]. Although the label dose of alglucosidase alfa is 20 mg/kg/2 weeks, it is currently known that higher dosing improves outcomes in children with IOPD [[8], [9], [10], [11]]. Therefore, we decided to treat our patient with a higher dose of 40 mg/kg every other week as the starting dose.

Although ERT has greatly improved survival in IOPD patients, immune reactions can reduce ERT's effectiveness and safety. It is important to note that approximately 50 % of IOPD patients develop IARs [12]. 5–14 % of patients treated with alglucosidase alfa develop allergic reactions involving ≥2–3 body systems, and around 1 % develop anaphylactic shock [5,12]. Regarding clinical studies with avalglucosidase alfa, in a pooled safety analysis 43.5 % of patients experienced hypersensitivity reactions, including 4.3 % of patients who reported severe hypersensitivity reactions and 1.4 % of patients who experienced anaphylaxis [13]. This is extremely important, as treatment with ERT is vital for IOPD patients.

Allergic hypersensitivity reactions are antigen-specific and can be IgE or non-IgE mediated. In these cases, skin tests and intradermal tests are usually positive [5]. General measures against immediate hypersensitivity reactions including premedication with antihistamines, antipyretics, and/or corticosteroids, short interruption of the infusion and slowed infusion rates have been successful in the management of IARs in patients receiving ERT. However, when these measures fail to prevent severe/recurrent reactions, desensitization is indicated, as ERT administration is crucial for patients with IOPD.

Here we present the case of an infant with IOPD who developed anaphylaxia to ERT with alglucosidase alfa involving three systems (urticaria, bronchospasm and angioedema) at 18 months of age. Given the life-saving nature of treatment with alglucosidase alfa, a desensitization protocol was prepared to ensure the safe administration of ERT.

Avalglucosidase alfa is an improved version of the old alglucosidase alfa molecule that has more mannose-6-phosphate residues in the surface and is able to enter in the myofiber with higher efficiency [14]. When the treatment was switched to avalglucosidase alfa, given the similarity of the two molecules and the positive intradermal skin test to avalglucosidase alfa in the patient, another desensitization protocol was developed for the new treatment administration (Table 1B). As the patient was suffering a clinical decline, although higher doses may be associated to higher immunogenicity [15], a decision was made to switch to avalglucosidase alfa at a dose of 40 mg/kg every other week.

Few reports of a desensitization protocol to ERT in a patient with IOPD have been published to date, being all of them to alglucosidase alfa [[16], [17], [18], [19], [20], [21], [22]]. In many cases half a dose was administered weekly [[16], [17], [18], [19], [20], [21], [22]]. In here, we share our protocol administering the full dose of 40 mg/kg in a reasonable infusion time.

## 4 Conclusion

Severe IARs to ERT are not frequent, but life-threatening hypersensitivity reactions may occur in some IOPD patients. This is of paramount importance, as ERT is vital for these patients. In this report we provide a desensitization protocol to alglucosidase alfa and, for the first time, a desensitization protocol to avalglucosidase alfa, both delivered in a reasonable time of less than 6 h, and without any further reactions in our patient.

## Consent

Written informed consent was obtained from the patient's parents for the publication of this case report. A copy of the written consent is available for review by the Editor-in-Chief of this journal on request.

## CRediT authorship contribution statement

**Miriam Gendive:** Writing – review & editing, Writing – original draft, Visualization, Methodology, Investigation, Formal analysis. **Teresa C. Delgado:** Writing – review & editing, Visualization, Methodology, Investigation, Conceptualization. **María Unceta:** Writing – review & editing, Methodology, Investigation. **Arantza Arza:** Writing – review & editing, Methodology, Investigation. **Beatriz Sordo:** Writing – review & editing, Methodology, Investigation. **Aritza Segurola:** Writing – review & editing, Methodology, Investigation. **Javier De Las Heras:** Writing – review & editing, Writing – original draft, Visualization, Validation, Supervision, Resources, Methodology, Investigation, Conceptualization.

## Declaration of competing interest

Javier de las Heras has received speaking and scientific consultancy fees from Sanofi.

## Data Availability

No data was used for the research described in the article.

## References

[1] Tarnopolsky M., Katzberg H., Petrof B.J., Sirrs S., Sarnat H.B., Myers K. (2016). Pompe disease: diagnosis and management. Evidence-based guidelines from a Canadian expert panel. Can. J. Neurol. Sci..

[2] Kishnani P.S., Hwu W.L., Mandel H., Nicolino M., Yong F., Corzo D. (2006). A retrospective, multinational, multicenter study on the natural history of infantile-onset Pompe disease. J. Pediatr..

[3] Kishnani P.S., Corzo D., Leslie N.D., Gruskin D., Van der Ploeg A., Clancy J.P. (2009). Early treatment with alglucosidase alpha prolongs long-term survival of infants with Pompe disease. Pediatr. Res..

[4] Kishnani P.S., Corzo D., Nicolino M., Byrne B., Mandel H., Hwu W.L. (2007). Recombinant human acid [alpha]-glucosidase: major clinical benefits in infantile-onset Pompe disease. Neurology.

[5] Gragnaniello V., Deodato F., Gasperini S., Donati M.A., Canessa C., Fecarotta S. (2022). Immune responses to alglucosidase in infantile Pompe disease: recommendations from an Italian pediatric expert panel. Ital. J. Pediatr..

[6] de Las Heras J., Cano A., Vinuesa A., Montes M., Unceta Suarez M., Arza A. (2021). Importance of timely treatment initiation in infantile-onset Pompe disease, a single-centre experience. Children (Basel).

[7] Kronn D.F., Day-Salvatore D., Hwu W.L., Jones S.A., Nakamura K., Okuyama T. (2017). Management of confirmed newborn-screened patients with Pompe disease across the disease spectrum. Pediatrics.

[8] Chien Y.H., Tsai W.H., Chang C.L., Chiu P.C., Chou Y.Y., Tsai F.J. (2020). Earlier and higher dosing of alglucosidase alfa improve outcomes in patients with infantile-onset Pompe disease: evidence from real-world experiences. Mol. Genet. Metab. Rep..

[9] Khan A.A., Case L.E., Herbert M., DeArmey S., Jones H., Crisp K. (2020). Higher dosing of alglucosidase alfa improves outcomes in children with Pompe disease: a clinical study and review of the literature. Genet. Med..

[10] Ditters I.A.M., Huidekoper H.H., Kruijshaar M.E., Rizopoulos D., Hahn A., Mongini T.E. (2022). Effect of alglucosidase alfa dosage on survival and walking ability in patients with classic infantile Pompe disease: a multicentre observational cohort study from the European Pompe consortium. Lancet Child. Adolesc. Health.

[11] Kishnani P.S., Kronn D., Suwazono S., Broomfield A., Llerena J., Al-Hassnan Z.N. (2023). Higher dose alglucosidase alfa is associated with improved overall survival in infantile-onset Pompe disease (IOPD): data from the Pompe registry. Orphanet J. Rare Dis..

[12] Myozyme (2025).

[13] Nexviadyme (2025).

[14] Bolano-Diaz C., Diaz-Manera J. (2022). Therapeutic options for the management of Pompe disease: current challenges and clinical evidence in therapeutics and clinical risk management. Ther. Clin. Risk Manag..

[15] Kishnani P.S., Kronn D., Brassier A., Broomfield A., Davison J., Hahn S.H. (2023). Safety and efficacy of avalglucosidase alfa in individuals with infantile-onset Pompe disease enrolled in the phase 2, open-label Mini-COMET study: the 6-month primary analysis report. Genet. Med..

[16] Rohrbach M., Klein A., Köhli-Wiesner A., Veraguth D., Scheer I., Balmer C. (2010). CRIM-negative infantile Pompe disease: 42-month treatment outcome. J. Inherit. Metab. Dis..

[17] El-Gharbawy A.H., Mackey J., DeArmey S., Westby G., Grinnell S.G., Malovrh P. (2011). An individually, modified approach to desensitize infants and young children with Pompe disease, and significant reactions to alglucosidase alfa infusions. Mol. Genet. Metab..

[18] Baruteau J., Broomfield A., Crook V., Finnegan N., Harvey K., Burke D. (2014). Successful desensitisation in a patient with CRIM-positive infantile-onset Pompe disease. JIMD Rep..

[19] Karagol I.H., Bakirtas A., Yilmaz O., Topal E., Kucukcongar A., Ezgu F.S. (2014). Desensitisation of the youngest patient with Pompe disease in response to alglucosidase alfa. Allergol Immunopathol (Madr).

[20] Gragnaniello V., Fecarotta S., Pecoraro A., Tarallo A., Catzola A., Spadaro G. (2019). Desensitization of two young patients with infantile-onset Pompe disease and severe reactions to alglucosidase alfa. Neurol. Sci..

[21] Toh T.S.W., Chong K.W., Goh A.E.N., Goh J.C.Y., Ting T.W., Tan E.S. (2022). Enzyme replacement therapy desensitization in a child with infantile onset Pompe disease. Asian Pac. J. Allergy Immunol..

[22] Ertoy Karagol H.I., Inci A., Terece S.P., Kilic A., Demir F., Yapar D. (2023). Long-term experience with anaphylaxis and desensitization to alglucosidase alfa in Pompe disease. Int. Arch. Allergy Immunol..

